# Food safety knowledge and practice of abattoir and butcher shop workers: a health risk management perspective

**DOI:** 10.1186/s42522-022-00070-1

**Published:** 2022-08-28

**Authors:** Daniel Teshome Gebeyehu, Habtam Tsegaye

**Affiliations:** School of Veterinary Medicine, Wollo University, Dessie, Amhara Ethiopia

**Keywords:** Food Safety, Knowledge, Practice, Abattoir workers, Butcher shop workers, Health risk perspective

## Abstract

**Background:**

Meat is rich in essential proteins and valuable nutrients for human health. Despite these benefits, it is a favorable medium for microbial growth and transmission to humans unless recommended safety procedures are followed. This research aimed to assess the level of knowledge and practice of the abattoir and butcher shop workers who were working in the meat value chain.

**Methods:**

The cross-sectional study design, using structured questionnaire interviews was used to assess the knowledge and practice of abattoir and butcher shop workers. A total of 226 randomly selected workers were participated in this study and bivariate logistic regression was used for data analysis.

**Results:**

Of 226 total participants, 46% were abattoir workers and 54% were butcher shop workers. Majority (88.9%) of the participants did not know about meat safety and 74.3% of the participants had insufficient practices. The cumulative knowledge of both abattoir and butcher shop workers was significantly associated (*p* = 0.001) with their knowledge of the presence of diseases causing agents in meat, the source of meat contamination, and the common critical points of meat contamination along the meat value chain. The cumulative practice of abattoir workers was significantly associated with their practices on daily meat transporting vehicle washing (*p* = 0.007), reducing meat contamination (*p* = 0.001), duration of animal fasting before slaughter (*p* = 0.039), cleaning of the animal body before slaughter (*p* = 0.002), cleaning material used in the abattoir (*p* = 0.003), disposal of abattoir waste (*p* = 0.002), and type of biosecurity measures used (*p* = 0.013). Similarly, the cumulative practice of butcher shop workers was associated (*p* = 0.001) with their practices of attracting customers, storing remaining meat from daily sales, and measures on contaminated food. Employment of the participants was significantly associated with both the cumulative knowledge (*p* = 0.007) and practice (*p* = 0.001) of the participants while the age of the participants was associated (*p* = 0.001) with only their cumulative practices.

**Conclusions:**

In general, the participants’ food safety knowledge and practice were unsatisfactory. As a result, the integrated food safety policy formulation in a One Health framework, and collaborative awareness creation among different food safety stakeholders were recommended.

**Supplementary Information:**

The online version contains supplementary material available at 10.1186/s42522-022-00070-1.

## Introduction

Meat is rich in essential protein and valuable nutrients for human health. Despite these benefits, it is a favorable medium of microbial growth and transmission to humans [1]. Especially, meat contaminations from meat handlers’ bodies, the hide of animals, the gastrointestinal system of the animals, and the meat processing environment are the common health risks for the meat consumers [2]. The food handlers’ poor knowledge about the food contaminants and good hygienic practices are the main factors for poor prevention and control practices of these contaminants [3–6]. The meat handlers, which include slaughterers, meat inspectors, transporters, meat processors, and butcher shop workers are expected to be knowledgeable on foodborne pathogens, temperature control, cross-contamination, and cleaning and sanitation activities.

Foodborne diseases are common public health hazards in both developed and developing countries regardless of their economic status and geographic locations [7]. The public health burdens of foodborne microbes are higher in developing countries [8] due to poor food handling and sanitation practices, inadequate food safety laws, weak regulatory systems, lack of financial resources to invest in protective equipment, and lack of education for food handlers [7]. The foods intended for human consumption; especially animal-origin food is most hazardous unless the food safety principles are employed [9]. Since meat is a highly perishable food stuffs [10] and the abattoirs and butcher shops are such labor-intensive working areas, the awareness, and level of training of the meat handlers regarding good hygienic management and the critical control points of the food chains are of great significance to mitigate the health risk of meat consumers [11].

During meat processing activities, the meat has the potential to be contaminated by pathogens. To produce safe and wholesome meat, the meat handlers should practice according to the food safety standards [12]. Such standards include good biosecurity practices, good manufacturing practices, good hygiene practices, and standard operating systems [13]. According to [14], there are more than 250 known food-borne diseases causing agents (bacteria, viruses, or parasites).

Hygiene problem is not only limited to slaughtering operations, but it is also associated with imprudent processing, marketing, and operating systems [3–6, 15, 16]. The knowledge and practices of the abattoir and butcher shop workers about food safety are therefore very crucial to eliminate the emerging and re-emerging zoonotic microorganisms and to produce healthy and wholesome meat for consumers.

Not only processed (cooked, roasted, stewed, and fried) meat, eating raw beef is commonly practiced throughout Ethiopia, but no written document is available about how this practice began. There is a verbal story that describes eating raw beef was began during wartime in which soldiers did not have access to fire and had limited time for cooking. Besides beef, eating raw meat from other animals is not common. The raw beef in Ethiopia is called "Kurt" in the Amharic language. Kurt is directly consumed without any process by mixing with hot paper and other locally prepared spices.

As presented by the international organization for animal health [16], the most prioritized bacterial hazards that can originate from raw meat are *Salmonella, Escherichia coli, Campylobacter, Shigella, and Listeria monocytogenes*. A pooled prevalence study showed that *Staphylococcus spp., Listeria monocytogenes, E. coli O157: H7,* and *Salmonella* were isolated from raw meat and meat products in Ethiopia and those bacteria are resistant to common antibiotics ceftriaxone, gentamicin, ciprofloxacin, and ampicillin in a variable degree of resistance [17]. The raw meat-eating behavior of many Ethiopians increases the vulnerability of consumers to meat-borne pathogens. Since the raw meat could not pass through different preparation processes (cooking, roasting, and radiation), the pathogens can pass directly to the consumers. The public health risk is remarkably high if these pathogens are antimicrobial-resistant [17].

Meat-eaters in developing countries including Ethiopia are affected by meat-borne pathogens. Food-borne diseases had 0.2, 0.1, and 1.3 disability-adjusted life year per year (DALY) per household in Gondar, Lalibela, and Debark cities respectively [18]. This is due to a lack of knowledge about good meat handling and improper food handling and hygienic practices. The knowledge and practice gaps of meat value chain workers can be due to a lack of food safety laws, weak enforcement of food safety rules, and lack of education for food handlers (abattoir, butcher shop, and meat processing workers). Since the food handlers’ level of knowledge and their practices are essential for policy formulation, awareness creation, ensuring consumers' access to safe food, and prevention of food contamination and disease occurrence, it was imperative to conduct this study along the meat value chain.

Therefore, the objectives of this study were:

- To assess the level of knowledge and practice of abattoir and butcher shop workers.
- To provide the necessary food safety information for awareness creations and policy proclamations

## Materials and methods

### Study area and population

**Fig. 1:**
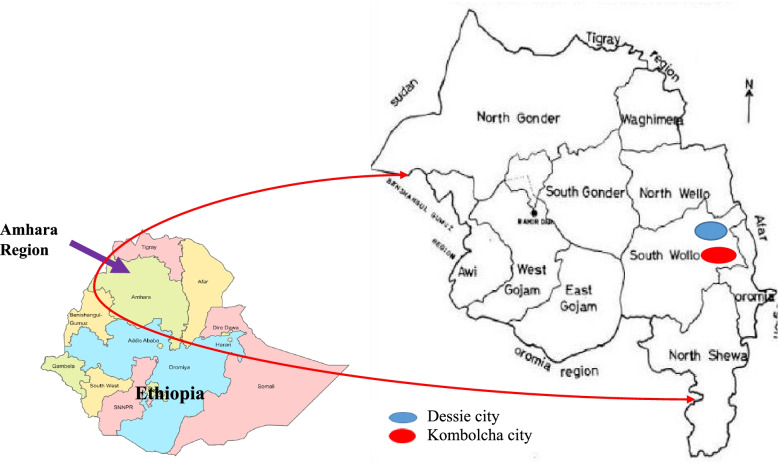
Location of study areas (Dessie and Kombolcha cities)

The target study population was abattoir and butcher shop workers in Dessie and Kombolcha cities. Dessie and Kombolcha cities are in the South Wollo Zone, Amhara regional state with the geographic coordinates of 11.1270° N, 39.6363° E, and 11.0849° N, 39.7292° E, respectively. The locations of Dessie and Kombolcha cities are indicated in Fig. 1.

These two cities are situated in the north-eastern part of Ethiopia, 376, and 401 km away from Addis Ababa (the capital of Ethiopia), respectively. According to the 2017 estimates, the population numbers were 102, 244 for Kombolcha city and 209, 226 for Dessie city [19]. Each city has one abattoir, which serves cattle and small ruminants’ slaughtering purposes. Slaughterhouse, and butcher shop workers including the meat inspectors (veterinarians and animal health workers), meat processors, meat sellers, meat transporters, and sanitary personnel (cleaners) were the source of information for this study.

### Study design

A cross-sectional study (a type of study used for investigating a situation at a point of time) was conducted from November 2020 to May 2021 in Kombolcha Elfora abattoir, Dessie city municipal abattoir, and butcher shops of the two cities. Face-to-face interviews were conducted using a structured questionnaire to collect information about the knowledge and practice of abattoir and butcher shop workers in both Kombolcha and Dessie cities.

Open-ended questionnaire items were employed for the sake of easily encoding and summarizing the response of the participants [20]. Since there was no validated pre-existing questionnaire in this research interest, the authors developed a new questionnaire. The close-ended questionnaire was validated using content validation methodology, which is effective and useful during new questionnaire development [21]. This validation was done by the experts invited from the food safety and public health fields and approved as the questionnaire items were effective to achieve the objective of the research interest. The questionnaire items were short, not exhaustive for filling, clear, and time efficient. One interview took around half an hour, and all the interviewees were interviewed during their tea break and right after completing their work.

The questionnaires were constructed in three sections. The first section was about the demographic characteristics of the respondents. Such as location, age, sex, educational levels, and employment status. The second section was about the participants’ knowledge of food safety, specifically about meat safety and hygiene. The last section was about the practice of respondents on meat hygiene and safety. In sections two and three there were questions that enabled the researcher to understand the respondents’ knowledge and practice, respectively. The questions in these sections included six knowledge-oriented questions and seven practice-based questions. Before the data collection, the questionnaires were pre-tested/validated on the butcher shop and abattoir workers in Hayk town. The pilot study for the validation of the questionnaires was done for two consecutive weeks.

### Sampling strategy

**Fig. 2:**
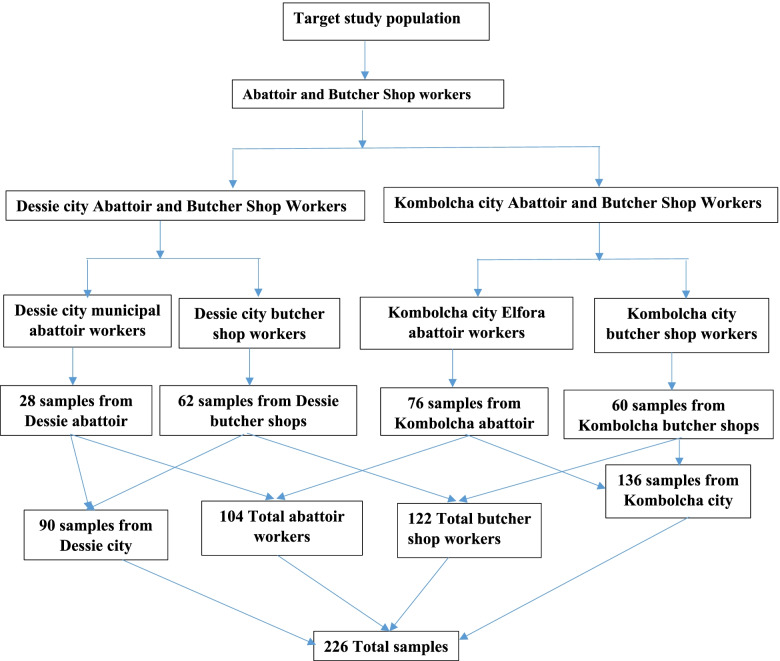
Samples proportion among Dessie and Kombolcha cities and abattoir and butcher shop workers

The abattoirs and butcher shops, located in both Kombolcha and Dessie cities were taken as a sampling frame. Since there was only one abattoir in each city, these abattoirs were directly sampled. All abattoir workers who were volunteers to be interviewed were invited for an interview. From both abattoirs, 104 total samples were collected. Since the abattoir in Kombolcha is an international export abattoir with larger number of employees, 73% of the participants were selected from it, and the remaining 27% were recruited from Dessie municipal abattoir (Fig. 2).

There were 99 butcher shops in Dessie and 35 in Kombolcha. From these shops 26 and 14 butcher shops were randomly selected in Dessie and Kombolcha cities, respectively. From each shop, workers were randomly selected for interview. The interview was continued until information saturation was attained. In both cities, a total of 122 butcher shop workers were interviewed. Workers who were aged < 18 were excluded from the interview and the rest were approved regardless of their educational levels, gender, sex, Employment status, and locations.

### Data collection and processing

Both knowledge and practice-oriented questions were presented to participants. The questions were related to the knowledge and practice of the respondents in their respective workplaces. As a result, some questions are specific to each working area (abattoir and butcher shop working areas). All knowledge-related questions were summarized into a single variable, which had two categories (knowledgeable and not knowledgeable) [22–25]. This variable was named as the cumulative knowledge of the participants that enables the researcher to associate with other predictor variables. In the same way, the cumulative practice was produced from the practice-oriented questions and the response of the participants were categorized into either sufficient practice or insufficient practice [22, 24, 25].

### Data analysis

After the data was collected and coded, it was administered in Microsoft excel 2013. For the sake of analyzing by logistic regression, the knowledge and practice variables and demographic data of the respondents were coded with numbers. During statistical analysis, the Microsoft excel recorded and coded variables were directly copy-pasted to the SPSS data sheet.

The dataset was composed of 20 and 19 categorical variables for abattoir and butcher shop workers, respectively. Based on Likert’s scale [25], dependent binary variables were produced for knowledge and practice, from each respective question/variable. The purpose of producing these two dependent variables was to assess the effect of each predictor variable on the knowledge and practice of the participants, using logistic regression. The participants who correctly answer all knowledge-related questions were categorized into knowledgeable and those who fail to answer one or more were grouped into not knowledgeable. The knowledge-related questions with “yes” answers were easily grouped into knowledgeable and “no” answers were grouped into not knowledgeable while the rest of the questions with correct answers were grouped into knowledgeable and incorrect answers were categorized into not knowledgeable. The participants who practiced according to the Ethiopian meat safety standards [26] were categorized as sufficient practice, and those who violated these meat safety standards were grouped as insufficient practice.

Logistic regression was used to see which explanatory variables were predictive for the result, knowledgeable or not knowledgeable, and sufficient practice or insufficient practice. The investigation of the participants’ knowledge and practices on meat safety was followed in three steps. The first step was assessing the relationship between potential predictor variables with the participants’ knowledge, and practice. Secondly, the relationship for the potential confounding effects was adjusted. Finally, the possibility of an interaction effect among the variables was considered.

To have initial insight into the structure of the data, cross-tabulations were used in SPSS. From this basic descriptive tool, it is possible to see the proportions of each response category, which were indicative of the level of participants’ knowledge and practice about meat safety in both abattoir and butcher shops.

After descriptive investigations using crosstabs, the association between the dependent binary variables (knowledge and practice) and each predictive variable was conducted. Likelihood ratio test (*LR* test), and probability values (*p*) were used to see the association between these dependent binary variables (knowledge and practices) and predictive variables (variables produced from each question). The *p*-value < 0.05 were taken as a statistically significant association between the predictor variables and the dependent binary variables. The effect levels of demographic variables on the knowledge and practice of the participants were shown by the odds ratio (*OR*). Wald’s chi-square test and probability value were used to see the association between demographic characteristics with the knowledge and practice of the participants.

## Result

### General description of the participants

From both study places (Dessie and Kombolcha), a total of 226 samples were collected from two kinds of business operators (Abattoirs and Bucher shops). The majority (60.2%) of the participants were from Kombolcha city and the remaining 39.8% were from Dessie city. Among the respondents, 104 (46%) were abattoir workers and 122 (54%) were butcher shop workers. The socio-demographic details of the participants are described in Table 1.

**Table 1:** Demographic information of both abattoirs and butcher shops workers

Variables	Categories	Number (%)		
		**Total (*n* = 226)**	**Abattoir workers (*n* = 104)**	**Bucher shop workers (*n* = 122)**
**Location**	Dessie	90(39.8)	28(26.9)	62(50.8)
	Kombolcha	136(60.2)	76(73.1)	60(49.2)
**Age**	18–30	99(43.8)	17(16.3)	82(67.2)
	31–40	82(36.3)	45(43.3)	37(30.3)
	> 40	45(19.9)	42(40.4)	3(2.5)
**Sex**	Male	138(61)	66(63.5)	72(59.0)
	Female	88(38.9)	38(36.5)	50(41.0)
**Educational level**	Illiterate	43(19.0)	32(30.8)	11(9.0)
	Primary	54(23.9)	27(26.0)	27(22.1)
	Secondary	92(40.7)	37(35.6)	55(45.1)
	Tertiary	37(16.4)	8(7.7)	29(23.8)
**Employment status**	Daily Laborers	43(19.0)	32(30.8)	11(9.0)
	Contracts	109(48.2)	27(26.0)	82(67.2)
	Permanent employee	74(32.7)	45(43.3)	29(23.8)

*n *number of samples, (Dessie, 2022), the numbers inside the bracket are the percentage of the participants and outside the bracket are the number of participants in each category

**Fig. 3:**
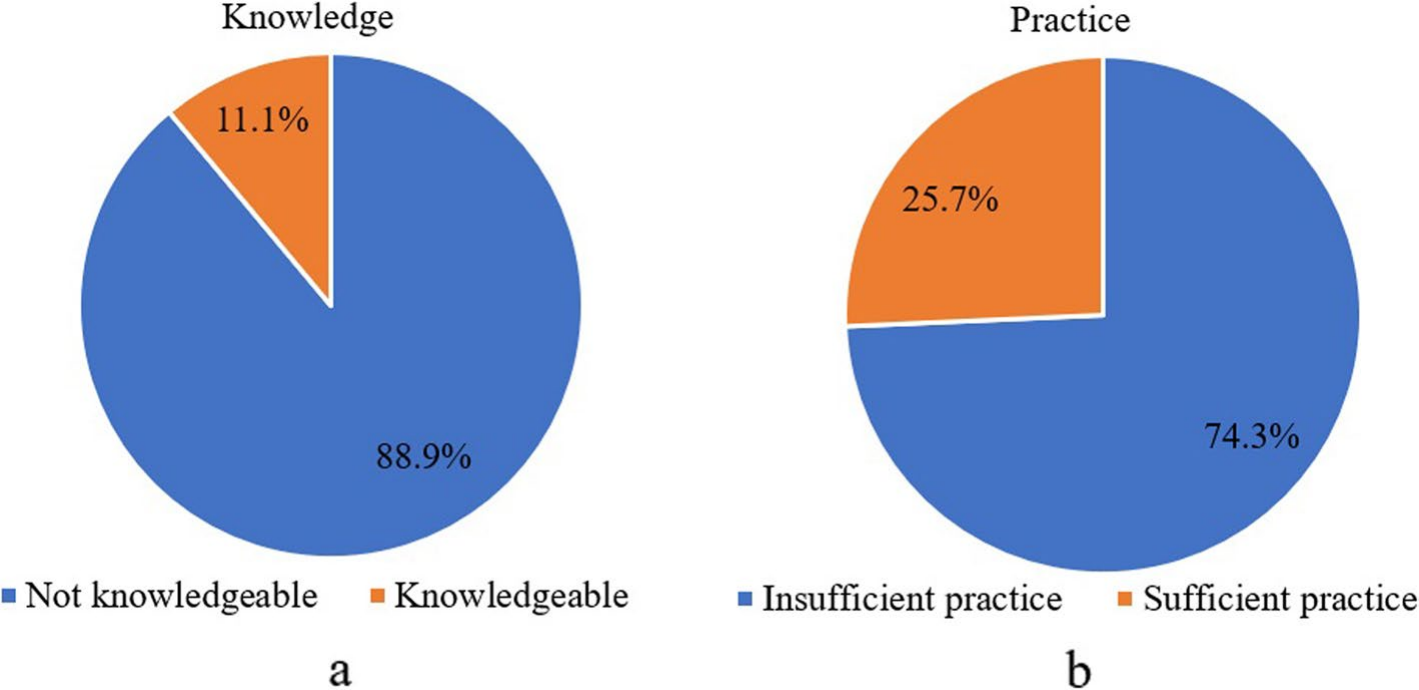
The knowledge (Fig. 3**a**) and practice (Fig. 3**b**) status of the abattoir and butcher shop workers toward meat safety

The majority (88.9%) of the participants were not knowledgeable about meat safety (Fig. 3a) and the majority (74.3%) of the butcher shop and abattoir workers’ practices towards meat safety were insufficient (Fig. 3b).

### The knowledge of the abattoir workers about food safety

The cumulative knowledge of the participants was significantly associated (*p* < 0.05) with each knowledge related variables, except the participants’ knowledge about specific diseases in meat (Table 2).

**Table 2:** The proportions of knowledge related questions and association of variables with the cumulative knowledge of abattoir workers

Variables/Questions	Responses	Number (%) (*n* = 104)	*LR* test score	*p* value
Meat can be a source of infection	Agree	86(82.7)	5.760	0.016
	Disagree	18(17.3)		
What diseases causative agent do you know in meat?	Bacteria	34(32.7)	25.305	0.001
	Parasite	23(22.1)		
	More than one causative agent (Parasite, Virus, and Bacteria)	47(45.2)		
Did you know where the source of contamination is?	Animals	46(44.2)	25.952	0.001
	Slaughtering area and equipment	34(32.7)		
	Personnel	10(9.6)		
	Animals, equipment, and personnel	14(13.5)		
Along the meat processing value chain, where can meat be contaminated?	In the abattoir	46(44.2)	63.105	0.001
	During transport	21(20.2)		
	In the butcher shop	19(18.3)		
	All abattoir, butcher, and transport	18(17.3)		
Do you know diseases that can originate from meat?	Yes	97(93.3)	2.101	0.147
	No	7(6.7)		
Can fasting of the animal reduce contamination?	Yes	89(85.6)	4.714	0.030
	No	15(14.4)		

*n *number of samples, *LR* *test score* likelihood ratio test, *p* *value* probability value, (Dessie, 2022), the numbers inside the bracket are the percentage of the participants and outside the bracket are the number of participants in each category

### The practice of the abattoir workers on meat safety

Like the knowledge of the abattoir workers, their summarized practices were significantly associated (*p* < 0.05) with all the practice-oriented questions/variables. The details of participants’ responses with their statistical descriptions are described in Table 3.

**Table 3:** The proportions of practice related questions and association of variables with the cumulative practice of abattoir workers

Variables/Questions	Responses	Number (%) (*n* = 104)	*LR* test score	*p*- value
What is your daily vehicle washing schedule?	Before transport	30(28.8)	12.225	0.007
	Before and after transport	55(52.9)		
	Once per day	15(14.4)		
	No specific time to wash	4(3.8)		
What are you doing to reduce meat contamination?	Washing by water	49(47.1)	13.045	0.001
	Cleaning and disinfecting with soap and bleach	12(11.5)		
	Avoiding contact with bare hands, walls, and unhygienic utensils	43(41.3)		
For how much time the animals fast before slaughter	> 24 h	17(16.3)	6.465	0.039
	< 24 h	74(71.2)		
	I do not know	13(12.5)		
Did you clean the body of the animals before slaughter?	Yes	62(59.6)	9.883	0.002
	No	42(40.4)		
What cleaning material did you use in your abattoir?	Water only	35(33.7)	11.524	0.003
	Water with soup	12(11.5)		
	Disinfectants	57(54.8)		
How do you dispose of abattoir’s waste?	Incineration and Burial	62(59.6)	9.883	0.002
	To the surrounding environment	42(40.4)		
What type of biosecurity measure did you use?	Changing room	24(23.1)	10.735	0.013
	Foot bath	35(33.7)		
	Washing room	22(21.2)		
	Nothing	23(22.1)		

*n *number of samples, *LR* *test score* likelihood ratio test, *p* *value* probability value, (Dessie, 2022), the numbers inside the bracket are the percentage of the participants and outside the bracket are the number of participants in each category

### The meat safety knowledge of the butcher shop workers

The questions delivered for butcher shop workers were different from that of abattoir workers. All 122 butcher shop workers in two study cities were evaluated with the same set of questions. The responses for each question and the percentage of the respondents for each response category are shown in Table 4. The *LR* test score and *p* values, which show the associations of each variable to the summarized knowledge of butcher shop workers are indicated in Table 4 as well.

**Table 4:** The proportions of knowledge related questions and association of variables with the cumulative knowledge of butcher shop workers

Variables/Questions	Responses	Percent (*n* = 122)	*LR* test score	*p*-value
Do you think that meat is a source of diseases causing agents?	Yes	66(54.1)	14.438	0.001
	No	56(45.9)		
What is the problem with cleaning your equipment with water only?	Improper dirt cleaning	19(15.6)	8.978	0.011
	Improper microbe removal	83(68.0)		
	No problem	20(16.4)		
What diseases causative agent do you know in meat?	Bacteria	71(58.2)	28.316	0.001
	Parasite	12(9.8)		
	More than one causative agent (Parasite, Virus, and Bacteria)	39(32.0)		
What are the sources of meat contamination across the beef value chain?	Working environment	24(19.7)	22.238	0.001
	Equipment	44(36.1)		
	Personnel	6(4.9)		
	Environment, personnel, and equipment	48(39.3)		
What is the problem if you did not use personal protective equipment (head cover, mask, boots, and gloves)?	Workers may be infected with foodborne diseases	98(80.3)	49.855	0.001
	Contamination of the meat from workers	18(14.8)		
	No problem	6(4.9)		
Is prevention of meat borne diseases possible?	Yes	107(87.7)	3.035	0.081
	No	15(12.3)		
What temperature setting is recommended for refrigeration of meat?	< 0 °C	32(26.2)	11.172	0.004
	< 4 °C	47(38.6)		
	No setting just on and off	43(35.2)		

*n *number of samples, *LR* *test score* likelihood ratio test, *p* *value* probability value, (Dessie, 2022), the numbers inside the bracket are the percentage of the participants and outside the bracket are the number of participants in each category

Except for the possibility of meat-borne disease prevention, all variables that were used for assessing the knowledge of butcher shop workers were significantly associated (*p* < 0.05) with the cumulative knowledge of participants. This indicates the level of knowledge in every single question is a significant factor in the cumulative knowledge of the participants about meat safety. As is presented in Table 4, the knowledge of the possibility of meat as a source of infection, the effectiveness of water for cleaning, the causative agent of meat-borne diseases, the source of contamination for meat, prevention of diseases from meat, the need of using personal protective equipment (PPE) and the use of appropriate refrigerator temperature was significantly associated (*p* < 0.05) with the general food safety knowledge of participants.

### The practice of butcher shop workers towards food safety

Except for participants’ exposure to contaminated meat (organoleptic contamination) and their customers’ preference for meat preparation, all other variables/questions were significantly associated *(p* < 0.05) with the cumulative practice of butcher shop workers. The percentage of the participants’ responses and its statistical association indicators (*LR* test score and *p* values) are described in Table 5.

**Table 5:** The proportions of practice related questions and association of variables with the cumulative practice of butcher shop workers

Variables/Questions	Responses	Number (%) (*n* = 122)	*LR* test score	*p* value
What did you do to attract customers?	Reduce price	61(50.0)	164.376	0.001
	Increase quality	49(40.2)		
	Increase sociability	12(9.8)		
How can you store the remaining meat from the daily sale?	Put in refrigerator	65(53.3)	91.827	0.001
	Put in the open air	8(6.6)		
	Cook and put it in for the next day	49(40.2)		
What did you do if you found grossly contaminated meat?	Cook and sell	32(26.2)	40.882	0.001
	Trim and remove	87(71.3)		
	Show to health workers	3(2.5)		
Did you find contaminated meat?	Yes	5(4.1)	0.000	0.994
	No	117(95.9)		
What type of meat preparation did your customers prefer?	Cooked meat	48(39.3)	4.727	0.094
	Raw meat	51(41.8)		
	Roasted meat	23(18.9)		

*n *number of samples, *LR test score* likelihood ratio test, *p* *value* probability value, (Dessie, 2022), the numbers inside the bracket are the percentage of the participants and outside the bracket are the number of participants in each category

### The inter-variable associations of participants’ meat safety knowledge and their practices

The knowledge of the participants on meat-borne disease causative agents was significantly associated *(p* < 0.05) with the cumulative practice of the participants. The abattoir workers’ knowledge about the meat-borne disease was significantly associated (*p* < 0.05) with the practice of storage mechanism of the remaining meat, the temperature setting of the refrigerator, and the problem of washing the equipment and abattoir only with water. The knowledge of the participants about the effectiveness of water for cleaning was associated with their practices on customer attraction. Their knowledge of the possibilities of foodborne disease prevention was associated with their practice in meat preparation. Likewise, the knowledge of the butcher shop workers about the recommended temperature setting of the refrigerator was associated (*p* < 0.05) with the practice of workers about the storage of remaining meat for daily sale.

The associations were not only between the cumulative knowledge of the participants and their predictor variables but also there were associations among the predictor variables/questions. The knowledge of meat contaminants was significantly associated (*p* < 0.05) with the knowledge of nodes of contamination and the purpose of using PPE. The knowledge of the participants about the importance of PPE and knowing the recommended refrigeration temperature had a relationship. In addition to these, the practice of the participants about customer attraction is associated with the practice of the workers' action when they face contaminated meat.

### The effect of demographic characteristics on the knowledge and practice of participants

The dummy variables were created, and their statistical associations were analyzed. As a result, the knowledge of daily laborers was significantly associated (*p* < 0.05) with contract and permanent workers (Table 6). In addition, the practice of participants aged 31–40 was significantly associated with 18–30. Likewise, the practices of male participants were significantly associated with females (Table 6). The practices of abattoir and butcher shop workers on meat safety were significantly associated (*p* < 0.05) with their employment type. The permanent employees were 0.29 times more knowledgeable and had 4.78 times more good practice than daily laborers while the participants aged 31–40 years had 7.25 times more good practice than the participants aged 18–30 years.

**Table 6:** The effect of demographic characteristics towards the knowledge and practice of abattoir and butcher shop workers on meat safety

Categorical predictor dummy variables	Knowledge				Practice			
	*Wald’s X*^*2*^*- test*	*p-value*	*OR*	*95% CI for OR*	*Wald’s X*^*2*^*- test*	*p-value*	*OR*	*95% CI for OR*
**Location**								
* Dessie vs. Kombolcha*	0.000	0.985	1.01	0.43–2.36	0.350	0.554	1.20	0.66–2.20
**Age**								
* 31–40 vs. 18–30*	0.052	0.820	1.15	0.34–3.89	12.411	0.001	7.25	2.41–21.81
* 41–62 vs. 18–30*	0.564	0.453	1.59	0.48–5.31	1.184	0.277	1.93	0.59–6.32
**Sex**								
* Female vs. Male*	0.302	0.583	1.27	0.55–2.93	3.962	0.047	1.87	1.01–3.38
**Educational level**								
* Primary vs. Illiterate*	1.238	0.266	0.27	0.03–2.71	0.610	0.435	1.57	0.51–4.82
* Secondary vs. Illiterate*	1.722	0.189	0.21	0.02–2.14	3.191	0.074	2.58	0.91–7.32
* Tertiary vs. Illiterate*	3.083	0.079	3.15	0.88–11.33	1.414	0.234	1.82	0.68–4.91
**Employment**								
* Contract vs. Daily*	5.438	0.020	0.09	0.01–0.68	3.104	0.078	2.50	0.90–6.93
* Permanent vs. Daily*	7.254	0.007	0.29	0.12–0.71	13.634	0.001	4.78	2.08–10.98
**Occupation**								
* Abattoir vs. Butcher*	1.114	0.291	0.64	0.28–1.47	24.616	0.001	7.09	3.27–15.36

*Wald’s X2− test*  Wald’s chi−square test,  *p−value* Probability value, *OR* Odds ratio, *95%* *CI* 95 percent confidence interval, (Dessie, 2022)

## Discussion

The number of participants from butcher shops was higher than in abattoirs, which were 122(54%) and 104(46%) respectively. This was due to a higher number of butcher shops than the abattoirs in the study areas (Dessie and Kombolcha cities). The result of this study showed that the majority (88.9%) of the abattoir and butcher shop workers were not knowledgeable about the meat safety issues. This indicates that the participants were not informed or trained about the proper food safety standards, which leads them to have food safety awareness gaps. Comparable with the current finding, 61.2%, 60%, and 70.6% of the respondents in Malaysia [27], Wollega [28], and Ghana [29] were not knowledgeable about the food safety, respectively. This means, that there was no, or low meat safety sensitization was done for the abattoir and butcher shop workers in all the above-mentioned study areas including the current study site. On the opposite, a study conducted in northwestern Ethiopia [30] showed that 79.1% of the meat handlers had good food safety knowledge. In addition to this, a study conducted in Ghana [29] showed that the butcher shop workers were knowledgeable in handwashing (98.7%), using gloves (77.9%), proper cleaning of the instruments (86.4%), and detergent use (72.8%). This difference might be due to differences in socio-demographic characteristics, community awareness, and food safety governance between the current study area and northwestern parts of Ethiopia and Ghana.

The current study indicated that 74.3% of the butcher shop and abattoir workers had insufficient food safety practices. This substantial insufficient food safety practices can affect the quality of meat in the study area which needs consecutive awareness creation interventions and integrated food safety regulation formulation and enforcement. Comparable with this study, the finding in Wollega [28] showed that 54.2% of food handlers had insufficient practices. Since the current study area and Wollega are found in the same country and governed by the same food safety regulations, it is expected to have similar food safety practices among workers. The participants’ insufficient practices in the present study might be due to a lack of knowledge on meat safety issues. On the contrary, the studies in Gondar (57.5%) [31], Malaysia (77.7%) [27], and Gondar (66.4%) [32], indicated that meat handlers had good food safety practices. This indicates that the meat handlers in Gondar and Malaysia had more awareness about meat safety than the abattoir and butcher shop workers in the current study areas. As a result, these variations might be due to differences in the enforcement of food safety rules and the awareness creation programs in these study areas.

The majority (82.7%) of the abattoir workers know that meat can be a source of infection. In addition to this, a high number (93.3%) of the participants did know at least one foodborne disease that could originate from meat. The understanding of abattoir and butcher shop workers about the pathogens originating from meat could increase the concern of workers in reducing meat contamination, but the practice of the workers on the ground is the opposite. This is comparable with the studies conducted in Northwest Ethiopia [30], Owerri [33], and Indonesia [34]. Even if the cumulative knowledge of the abattoir workers was not sufficient, they had a satisfactory level of understanding of meat-borne diseases. To prevent foodborne diseases, the majority (47.2%) of the abattoir workers cleaned the equipment and the abattoir with water only (without disinfection). Cleaning with water, without the help of disinfectants or detergents is not effective in detaching pathogens from food processing surfaces. This agrees with the findings reported from Western Kenya [35], Gondar [36], Bishoftu [37], and Gondar [31]. This might be because of the workers’ insufficient understanding of the low effectiveness of water for cleaning. It is recommended that cleaning is effective by using detergents, soaps, and disinfectants in addition to water. Forty percent of the abattoir workers were disposing of the abattoir wastes in the surrounding environment. This insufficient practice of the abattoir workers was alike to the study done in Western Kenya [35], Bishoftu [37], and Wollega [28]. These results are an indication of the participants’ knowledge gap on the re-circulation of the diseases from the environment to the meat or the low environmental sanitation regulation enforcement.

The majority (80.3%) of the butcher shop workers know that PPE (head-cover, masks, boots, gowns, and gloves) are used for personal prevention than the prevention of meat contamination. This indicated that the workers understood that they can be affected by pathogens from meat and failed to know that meat can be contaminated by them. On the opposite, the studies conducted in Aden [38], and Bishoftu [37] indicated that the body of the butcher shop workers especially their hands and the equipment they use were highly contaminated with pathogenic microbes, which might have the opportunity to pass to meat. As a result, the butcher shop workers only know the one-way benefit of the PPE, they did not know that it can be useful to prevent the transmission of microbes from workers to meat. Since the carcass inside the cattle is sterile, contamination is usually from intestinal content and dirty skin during dressing [39–42] due to post-slaughter management problems.

A substantial number (87.7%) of the participants know that the prevention of meat contamination was possible, but their practices were imprudent, which were in the way of aggravating the emergence of foodborne diseases. Only 2.5% of the butcher shop workers call meat inspectors when they find contaminated meat while the majority (71.3%) of the workers were trimming and removing the improper part of the meat by themselves. Standing on these findings, the insufficient meat safety practice is not only because of the knowledge gap but also due to limited or no follow-up of the food safety regulators. Alike the current study, the studies that were done in Northwest Ethiopia [30], Western Kenya [35], Owerri [33], and Jigjiga [7] showed that butcher shop and abattoir workers’ practices were the opposite of their knowledge about food safety. A high number (41.8%) of the butcher shop workers had customers whose eating preference was raw meat. This non-desired eating behavior (raw meat-eating practice) of the butcher shop customers could aggravate the emergence of foodborne and drug-resistant microbes from meat [18,40,43]. Since the food safety follow-up, environmental sanitation regulation enforcement, and the awareness level of the abattoir and butcher shop workers were insufficient, the consumers who are eating raw meat from these abattoirs and butcher shops are at high health risk.

The cumulative knowledge of both abattoir and butcher shop workers was significantly associated (*p* = 0.0001) with their knowledge of the presence of diseases causing agents in meat, the source of meat contamination, and the common critical points of meat contamination along the meat value chain. In line with this finding, the study reported from Wollega [28], and Ghana [29] indicated that the possibility of meat as a source of infection, the knowledge of specific diseases in meat, the knowledge of critical control points along the meat value chain, and knowledge about the source of contaminations was associated with the knowledge of abattoir workers. All butcher shop workers' knowledge-related variables, except the knowledge of the participants about foodborne disease prevention, were significantly associated (*p* < 0.05) with the cumulative knowledge of the participants (Table 4). This indicated that the level of knowledge in every single question is a significant factor in the cumulative knowledge of the participants about meat safety issues. This finding agrees with the finding of the studies conducted in Ghana [29], and Western Kenya [35]. In this study, the butcher shop workers' knowledge about the use of PPE was significantly associated (*p* < 0.05) with the cumulative knowledge of workers. Contrary to this finding, the study in Gondar [36] showed that meat handlers’ knowledge was not associated with their PPE using practices.

The cumulative practice of abattoir workers was significantly associated with their practices on daily meat transporting vehicle washing (*p* = 0.007), reducing meat contamination (*p* = 0.001), duration of animal fasting before slaughter (*p* = 0.039), cleaning of the animal body before slaughter (*p* = 0.002), cleaning material used in the abattoir (*p* = 0.003), disposal of abattoir waste (*p* = 0.002), and type of biosecurity measures used (*p* = 0.013). This means the cumulative food safety practice of abattoir workers are influenced by the individual practice of these variables. Like this finding, the cleaning practice of the workers, washing practice of equipment, the cleaning material used in the food value chain, waste disposal practice of the meat handlers, and biosecurity practice of workers, were associated (*p* < 0.05) with the cumulative practice of the participants in Ghana [29], Gondar [30], Wollega [28], and Western Kenya [35].

The cumulative practice of butcher shop workers was associated (*p* = 0.001) with their practices of attracting customers, storing remaining meat from daily sales, and measures taken on contaminated food. The imprudent practices of butcher shop workers in attracting customers, storing remaining meat for daily sale, and type of measures on contaminated food were the aggravating factors for the increased insufficient food safety practices. As indicated by the studies conducted in Wollega [28], Ghana [29], and Northwest Ethiopia [30], the practices of customer attraction, the storage practice of daily leftover meat, and the workers’ actions during contaminated meat exposure was associated with the cumulative practice of the meat handlers.

As the intervariable’ association indicated, there were associations among different predictor variables of knowledge and practice of participants. These inter-variable associations could have a confounding effect on the association of each predictor variable and the cumulative knowledge and practice of workers. Based on the inter-variable associations, the knowledge of abattoir and butcher shop workers about meat-borne diseases was significantly associated (*p* < 0.05) with the cumulative practice of the respondents. Alike this finding, the research findings in Gondar [32], Malaysia [27], Jimma [43], and Gondar [31], were showed that the awareness of food chain workers about foodborne diseases had an association with the practice of workers.

The knowledge of the participants on the meat-borne diseases was significantly associated (*p* < 0.05) with the practice of storage mechanism of the remaining meat, the temperature setting of the refrigerator, and the problem of washing only with water. This means the cumulative knowledge of workers is influenced by the knowledge of workers on each predictor variable. This was in line with the study done in Gondar [31], Wollega [28], Gondar [32], and Kenya [35]. As indicated in Table 5, butcher shop customers had different meat preparation preferences including raw meat preparation. The meat preparations (cooking and roasting) are methods of foodborne disease prevention [1,14]. As a result, the meat preparation practices of the butcher shop workers are depending on their knowledge about the potentiality of preparation methods in reducing foodborne diseases [17, 36]. The knowledge of the butcher shop workers about the recommended temperature settings of the refrigerator for different food items was associated *(p* < 0.05) with the practice of workers about the storage of remaining meat from daily sales. Comparable with this finding, the study in Western Kenya [35], Wollega [28], and Nairobi [44] showed that the butcher shop workers who had poor knowledge about refrigeration preservation mechanisms did not store their food in the refrigerator. As a result, the storing practice of leftover meat depends on the workers’ knowledge of the benefits of refrigeration, the accessibility of refrigerators, and the presence of sufficient electric power.

Except for the employment type of the participants, all the demographic characteristics did not have an association with the knowledge of the participants (Table 6). The employment type of participants had a significant effect on their knowledge of the participants. Based on this analysis, contract workers and permanent employees were 0.09 and 0.29 times more knowledgeable than daily laborers, respectively. This might be due to the short stay of the daily laborers in the abattoirs and butcher shops which inhibits them from having enough experience with the meat safety issues. This finding was in line with the finding in Gondar [32], and northwest Ethiopia [30] with the odds of 1.97 and 1.95, respectively. Not only this, a study conducted in DRC by [45] showed that there was an association between the type of work and the knowledge of the participants.

The age, sex, employment type, and occupation of the participants were associated *(p* < 0.05) with the practice of the participants. Based on this, the participants aged 31–40 years had 7.25 times more sufficient practice than the participants aged 18–30 years. The reason for this effect might be due to the exposure of medium-age groups to updated information by using social media or school-based food safety awareness creation programs. On the opposite to this finding, a study conducted in Gondar [33] showed that the age of the abattoir and butcher shop workers was not associated with the practice of the workers, but the result was supported by other research findings conducted in Gondar [31] and Malaysia [27]. The male participants had 1.87 times more sufficient meat safety practices than the female participants. The findings in Gondar [36], and DRC [45] were contrary to the current study, but the finding is in line with the studies done in Gondar [32], Mekelle [11], and Gondar [31] with the odds of 1.69, 1.72 and, 0.62, respectively. Permanent employees had 4.78 times more sufficient food safety practices than daily laborers. Since the permanent workers are food safety professionals, they might correctly act the food safety protocols than the daily laborers. The abattoir workers had 7.09 times sufficient meat safety practice than the butcher shop workers. This finding was comparable with the research finding in Addis Ababa [46] with the odds of 2.4.

### Conclusion and recommendation

The finding of the present study showed that the abattoir and butcher shop workers’ meat safety knowledge and practice are not sufficient. The participants were knowledgeable and had prudent practices on some meat safety issues, but their cumulative food safety knowledge and practices were unsatisfactory. These meat safety knowledge and practice gaps are the driving factors for unhygienic meat supply to consumers. In this context, the health risk level of meat consumers is expected to be high, and it is proposed for further investigation. The knowledge and practice gaps of abattoir and butcher shop workers are an indication of awareness creation gaps by the food business operators (abattoir and butcher shops) or by the food safety regulators. As a result, integrated awareness creations on the personal protective equipment usage, the biosecurity measures along the meat value chain, the transmission of public health hazards from meat to humans, meat contamination prevention, meat storage, and processing techniques, critical, control points of meat contamination, recommended cleaning materials along the meat value chain, and the general food safety guidelines are recommended. The food safety regulators are also recommended to formulate and enforce food safety regulations along the meat value chain in an integrated One Health framework. The health burden of meat-borne pathogens, the raw beef consumers’ health risk, the microbial contamination/load assessment along the beef value chain, and the attitude of the abattoir and butcher shop workers to change the current meat safety knowledge and practice are further studies of One Health.

### Limitations

The study might be liable to social desirability and recalling bias. In addition, the nature of the study design (cross-sectional) can influence the cause-and-effect relationship between the predictor variables and the dependent binary variables (knowledge and practice) of the butcher shop and abattoir workers. An intervention-based study method has a potential impact to overcome these limitations.

## Supplementary Information

**Additional file 1:** **S1 Excel.** The full data set that used to analyze the knowledge and practice of abattoir and butcher shop workers: The sheet represented by “abattoir workers” is the data set used to analyze the knowledge and practice of abattoir workers and the same for the sheet “butcher shop workers”. In sheet 3, the name of each variable is described

## Data Availability

The data used to support the findings of this study are included in the article in the frequency table. In addition to this, the whole data set used to analyze the food safety knowledge and practice of abattoir and butcher shop workers are attached in the supporting documents section (S 1). The data set is presented in an excel file leveled in 3 sheets.
